# Reproduction method for dried biomodels composed of poly (vinyl alcohol) hydrogels

**DOI:** 10.1038/s41598-018-24235-z

**Published:** 2018-04-10

**Authors:** Yasutomo Shimizu, Narendra Kurnia Putra, Makoto Ohta

**Affiliations:** 1 Institute of Fluid Science, Tohoku University 2-1-1, Katahira, Aoba-ku, Sendai, Miyagi Japan; 2 Graduate School of Engineering, Tohoku University 6-6, Aramaki-aza-aoba, Aoba-ku, Sendai, Miyagi Japan

## Abstract

Models mimicking the realistic geometries and mechanical properties of human tissue are requiring ever-better materials. Biomodels made of poly (vinyl alcohol) are particularly in demand, as they can be used to realistically reproduce the characteristics of blood vessels. The reproducibility of biomodels can be altered due to dehydration that is observed after long periods of usage. In order to improve their usability, one should consider the method used to reproduce them; however, few studies have reported a method reproduce biomodels. This study proposes a novel reproduction method for biomodels that allows them to quickly and easily reproduce their geometric and mechanical properties. Specimens of the dried biomodels were reformed through immersion in temperature-controlled water. Our results show that water at 35 °C can be effective to reproduce both the geometric and mechanical properties of the specimens. X-ray diffraction (XRD) measurements revealed that water immersion can reform the crystal structure of the pre-dried specimens, and images obtained using micro-computed tomography acquisition show that the geometry of the specimens can be reformed by water immersion without introducing any defects. These results indicate that the proposed method can lead to high reproducibility of both the original geometric and mechanical properties of the dried biomodels.

## Introduction

Interventional treatments have become much more widespread over the last ten years, and this has necessitated the development of appropriate training tools for medical practitioners^1^. *In vitro* models, which can accurately mimic the geometry of human tissue, are considered to be important tools for this purpose; an example of this is a blood vessel model that was developed and used to train neurosurgeons in the use of catheters^1^. Nowadays, the *in vitro* models used to train personnel in the use of catheters require the use of materials that more closely resemble the actual geometry, mechanical properties (such as stiffness and friction), and transparency of blood vessels^2,3^.

In order to meet the requirements for such *in vitro* models, biomodels made of poly (vinyl alcohol) hydrogel (PVA-H) materials have been developed; this is because PVA-H can be used to closely resemble both the geometries and mechanical properties of human tissue^4–6^. In this study, PVA-H was prepared using a dimethyl sulfoxide (DMSO)-water solvent^4,6,7^; the high transparency of the PVA-H created in this way allowed for direct observations to be made without requiring the use of X-ray imaging techniques^1^.

Several methods have been proposed for the fabrication of biomodels, because different methods can result in, among other things, different mechanical properties and transparencies of models as well as different fabrication times. The fabrication method chosen for producing such models should be able to reproduce the mechanical properties and geometries of human tissue as closely as possible, and its fabrication time should be short, so that a lot of models can be made. PVA-H is often produced through freeze–thaw cycle and cast-dried methods^8^; these methods are based on the physical bonds that are made within the crystal structure of PVA-H. Kudo *et al*., however, revealed that the chemical structures and water content utilized in the drying and re-swelling phases of freeze–thaw cycles can influence the characteristics of the hydrogels that are produced by this process^9^. Different fabrication methods can also influence the crystal structure of PVA-H, which means that even if the same drying and re-swelling processes are used, the characteristics of the PVA-H variants produced will be different. A cast-dried method recently developed by Otsuka *et al*.^10^ was found to produce PVA-H with high transparency that possessed the Young’s modulus of blood vessels^10^. Although the fabrication process was simple, this method required a long time (one week or more) to complete the entire specimens. As described previously, the short time to fabricate a model depicts various advantages because it can produce a higher number of models.

If PVA-H can maintain its geometry and mechanical properties after use, then PVA-H biomodels can be used repeatedly; if the solution in PVA-H evaporates over time, however, then the mechanical properties of PVA-H will likely change. Additionally, the PVA-H specimens can shrink and expand from their original geometries based on their water content. In order to prevent this from occurring, PVA-H should be usually preserved with submerged it on a preservative liquid. However, these preservation methods require the strict control of storage and treatment conditions while ensuring that the conditions in the hydrogel, such as the water content, do not change. One alternative storage method involves drying the hydrogels, as it makes them easier to handle and transport^11,12^. In earlier studies, the mechanical properties of dried PVA-H specimens were analyzed from a chemical viewpoint, such as by studying their crystallization behaviors^7,8,13,14^. However, the influence that drying and reformation processes have on the characteristics of biomodels is unclear due to the dynamic behaviors of these models; as such, the characteristics cannot be determined until after a treatment process for preservation has been performed. Therefore, a method is required to represent the geometry and the mechanical properties of realistic blood vessels while the models are being used.

The purpose of this study was to develop a simple reproduction method for the dried PVA-H biomodels in order to maintain the characteristics of the models for a long time. Alterations of the geometric and mechanical properties of the biomodels were measured in order to evaluate the effectiveness of the drying process. The weight, Young’s modulus, and dynamic viscoelasticity of PVA-H specimens were measured in order to investigate both how these mechanical properties changed due to drying and reformation and the effectiveness of the reformation method used in this study. X-ray diffraction (XRD) measurements were performed in order to investigate the crystal structure of the specimens in order to support the discussion about the changes in weight and Young’s modulus. Changes in the geometry of tube models (created using PVA) caused by the drying and reformation processes were measured using images acquired by micro-computed tomography (micro-CT) imaging.

## Materials and Methods

### Preparation of PVA-H

PVA powder (JF-17, DP = 1700, SV = 99 mol%, Japan Vam & Poval Co. Ltd, Japan) was dissolved in a solvent mixture of distilled water and DMSO (20/80 w/w, Toray Fine Chemicals Co. Ltd, Japan). Concentrations of 15 wt% and 17 wt% of PVA to the solvent were used, because these concentrations have been found to result in realistic mechanical properties of blood vessels being obtained in PVA-H blood vessel models^4^. The PVA solution was stirred for 2 h at 100 °C before it was cooled down to 40 °C and poured into a mold; it was then stored at −30 °C for 24 h in order to promote the gelation of the PVA solution. Molds with a diameter of 35 mm and a depth of 7 mm were prepared for the XRD measurements, and molds with dimensions of 8 mm × 1 mm × 50 mm were prepared for the tensile tests. Several PVA-H specimens were immersed in water for 24 h so that the DMSO in the solvent mixture would be replaced by water. The conditions used to develop the different PVA specimens are summarized in Table 1. The conditions in Table 1 were determined to discuss the influences of the concentration of PVA, the remaining amount of DMSO, drying states on the structure, and the mechanical properties of PVA-H specimens. The summary of the discussion points and samples for each test was described in Table 2.

**Table 1:** Conditions used to create different PVA-H specimens.

Sample code	Concentration of PVA [wt%]	w/ or w/o DMSO	Drying condition
(a)	17	w/	Before drying
(a*)	17	w/o	Before drying
(b)	15	w/	Before drying
(b*)	15	w/o	Before drying
(b**)	15	w/o	Dried 8 h
(b***)	15	w/o	Dried 8 h and reproduced 3 h

**Table 2:** Summary of the discussion points and sample codes for the tests performed in this study.

Test	Discussion point	Sample code
Weight ratio measurement	Influence of DMSO on drying time	(b), (b*)
Tensile test	Influences of DMSO, drying, and reformation on elasticity	(b), (b*), (b***)
Viscoelastic measurement	Influences of drying and reformation on dynamic mechanical properties	(b), (b***)
XRD	Detailed discussion of specimen structures	all
Micro-CT	Influence of drying and reformation on the model geometry	(b*), (b***)

### Weight measurements

A test was performed so that the behavior of the PVA-H specimens during drying could be investigated. The specimens were prepared as *per* conditions (b) and (b*) in Table 1. A hot-air drying method was used in this study. Drying methods are a simple and effective way to preserve many chemical specimens and these methods simply evaporate the water in a specimen *via* a stream of air^12^. The PVA-H specimens were dried at 40 °C and below 30% humidity through the use of an incubator (LTI-1001SD, Tokyo Rikakikai Co. Ltd., Japan); the change in weight of the specimens due to this process was measured and evaluated using Eq. 1, in which *W*, *W*_*t*_, and *W*_0_ represent the weight ratio, the weight at the time of a measurement, and the weight at the start of the experiment, respectively:

1 $$W=\frac{{W}_{t}}{{W}_{0}}$$

A test evaluating the reformation of the specimens was also performed; it was performed after the specimens had been dried, and it was conducted in order to check how well the mechanical properties of the pre-dried specimens were retained. The dried PVA-H specimens were immersed in temperature-controlled water; the temperature was controlled, as the temperature of the water used has been found to be a critical parameter for the swelling of hydrogels^15^. At the start of this test, every specimen was dried for 8 h using the incubator, based on the result of the drying test. After drying, the four specimens were immersed in water at temperatures that were 25 °C, 30 °C, 35 °C, and 40 °C below the melting point of PVA-H, respectively. In this condition, the weights of the specimens were measured until they had stabilized.

### Tensile tests

Following the weight measurements, tensile tests were performed on each specimen. The elasticity of every specimen, both before drying and after reformation, was evaluated by measuring their Young’s modulus using a uniaxial tensile tester (EZ-S, Shimadzu Co. Ltd., Japan); this tester recreated the dynamic deformations that occur due to pressure from pulsations within the human body, such as vessel deformation. The specimens used were prepared as per the conditions (b), (b*), and (b***) listed in Table 1, and the conditions of the tensile tests were based on those that were used in a previous study^16^. The distance between the top and bottom of the clamp used in the tests was set to 40 mm, and every specimen was stretched at a constant speed of 20 mm/min until a strain of 1.0 was reached, and then the specimen was returned to its original length under a strain of 0.0. This cycle was repeated three times; the Young’s modulus of every specimen was calculated using Eq. 2, which is based on Hooke’s law and was determined at a strain of 0.5 during the third cycle:

2 $$\sigma =E\varepsilon $$

In this equation, *σ*, *E*, and *ε* represent the stress, Young’s modulus, and strain parameters, respectively.

### Dynamic viscoelasticity measurements

The dynamic viscoelasticity of the specimens both before drying and after reformation were evaluated by measuring the complex viscoelastic modulus G* using a dynamic mechanical spectrometer (DMS-6100, SII NanoTechnology Inc., Japan); this would determine the minute deformations in the specimens. The dynamic storage modulus, *G′*, and the dynamic loss modulus, *G*″, were based on G* and were calculated using Eqs 3–5:

3 $${G}^{\ast }=\frac{S}{\gamma }$$

4 $$G^{\prime} =|{G}^{\ast }\,\cos \,\delta |$$

5 $$G^{\prime\prime} =|{G}^{\ast }\,\sin \,\delta |$$

In these equations, *S*, *γ*, and *δ* represent stress, strain, and the phase angle between the stress and strain, respectively. 8 mm × 8 mm × 1 mm specimens were prepared using the conditions (b) (used as a reference) and (b***) listed in Table 1. Dried versions of these specimens were immersed in water at 25 °C, 30 °C, 35 °C, and 40 °C. The specimens were also measured before drying so as to act as a reference point. Small amplitude (10 ± 0.5 *μ*m) sinusoidal oscillations at a frequency of 1 Hz were applied to the specimens along with a temperature of 23 °C based on the method published in a previous paper^4^.

### XRD measurements

θ*–*2θ measurements were performed using an X-ray diffractometer (SmartLab, Rigaku, Co. Ltd., Japan) in order to identify the crystal structure of the PVA-H specimens and facilitate the discussion on how the drying and reformation processes affected the mechanical properties of the samples. The specimens were prepared using the conditions outlined in Table 1. The conditions used for the X-ray measurements were as follows: voltage of 45 kV, current of 200 mA, excitation wavelength of 0.154 nm, and 2θ angles of 5–90° (intervals of 0.01°). The specimens (a) and (b) were covered by a sheet of polypropylene (PP) during the measurements to prevent from DMSO evaporation in the diffractometer. Time-lapse measurements of the PVA-H specimens were also carried out so that the details of the changes in the crystal structures could be discussed. The specimens used in the time-lapse measurements were produced as per conditions (b*) and (b***) in Table 1, and every measurement was observed after 30 min, 2 h, 4 h, and 6 h each.

### Image acquisition

Image acquisition was performed using micro-CT imaging (ScanXmate-D180RSS270, Comscantecno Co. Ltd., Japan), and the images were captured in order to evaluate the influence that the drying and reformation processes had on the geometries of tube models. A realistic tube model made from PVA-H was developed; the model resembled a human carotid artery, and it was prepared using a gypsum mold with a 15 wt% PVA solution. The PVA solution was coated on the surface of the mold by manual painting, and the PVA-H in the models was produced by the gelation of the PVA solution and the removal of the gypsum mold. The PVA-H was produced using the fabrication method described in “Preparation of PVA-H”. Images of the models both before drying and after being immersed in water (which took place three weeks after the drying process) were used to evaluate if any defects, such as cracks, occurred in the reformed models.

The X-ray conditions used for the image acquisition were a voltage of 100 kV and a current of 100 *μ*A. The parameters used for the image resolutions were as follows: magnification ratios of 2.12 and 2.54 for the straight and realistic vessel geometries, respectively, spatial resolutions of 60 and 50 mm for the straight and realistic geometries, respectively, and an image acquisition rate of 1800 during a 360° rotational acquisition. The detector in the micro-CT imaging system had a resolution of 1856 × 1472 pixels. After image acquisition was performed, the images were reconstructed using a piece of image processing software (coneCTexpress, White Rabbit Corporation, Japan). Three-dimensional (3D) images were developed using the reconstructed images. The thicknesses of the PVA-H models were measured using a different piece of image processing software (Fiji ver. 1.0, a distribution of ImageJ, National Institute of Health, USA).

## Results

### Weight measurements

**Figure 1:**
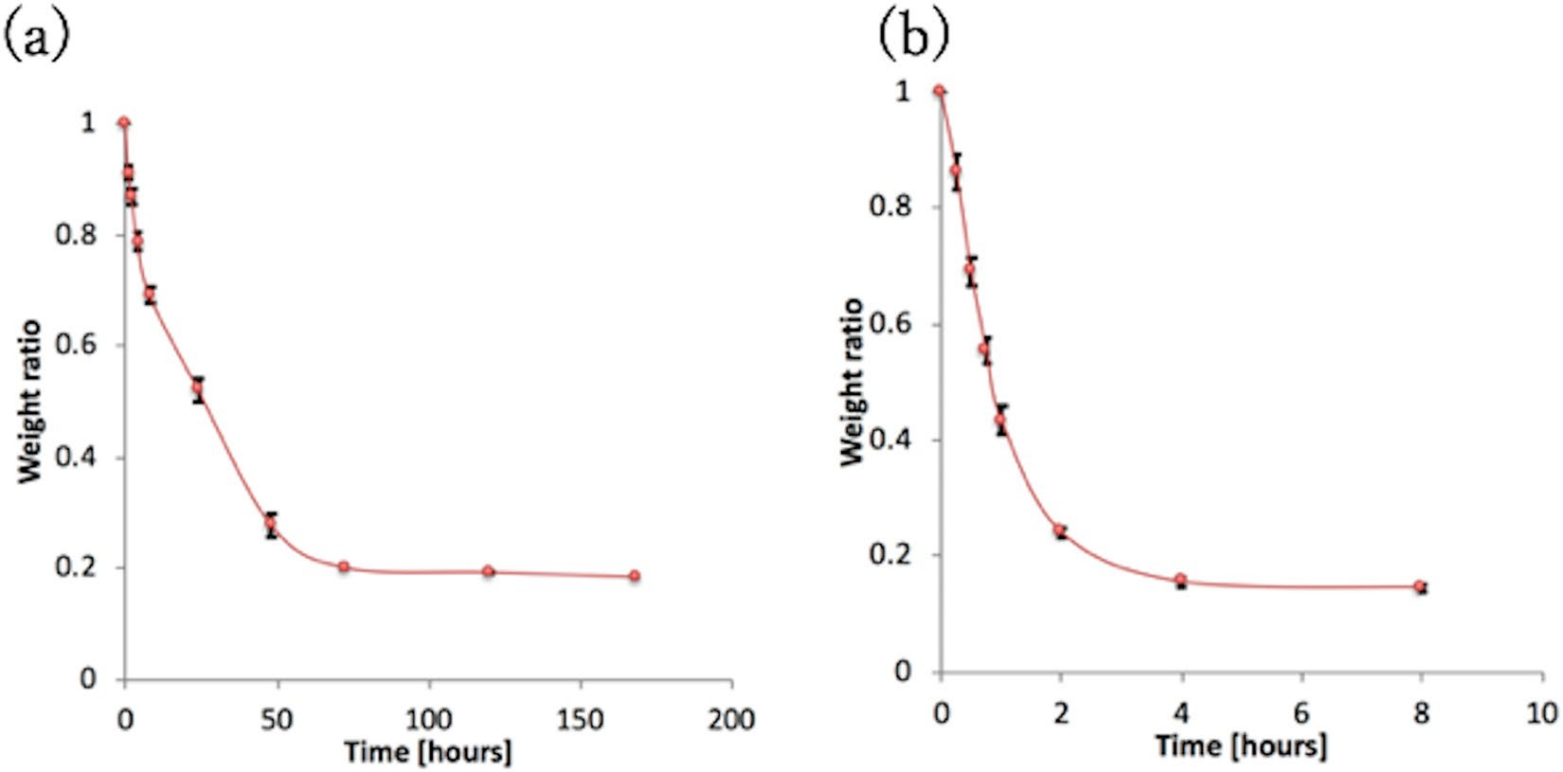
Change in the weight ratio of the PVA-H specimens as a function of the time taken for drying. A weight ratio of 1 means that the weight of the specimen is the same as before drying. (**a**) shows the results for PVA-H specimens that contain DMSO, and (**b**) shows the results for those specimens that did not contain DMSO. (mean ± SD, n = 3).

**Figure 2:**
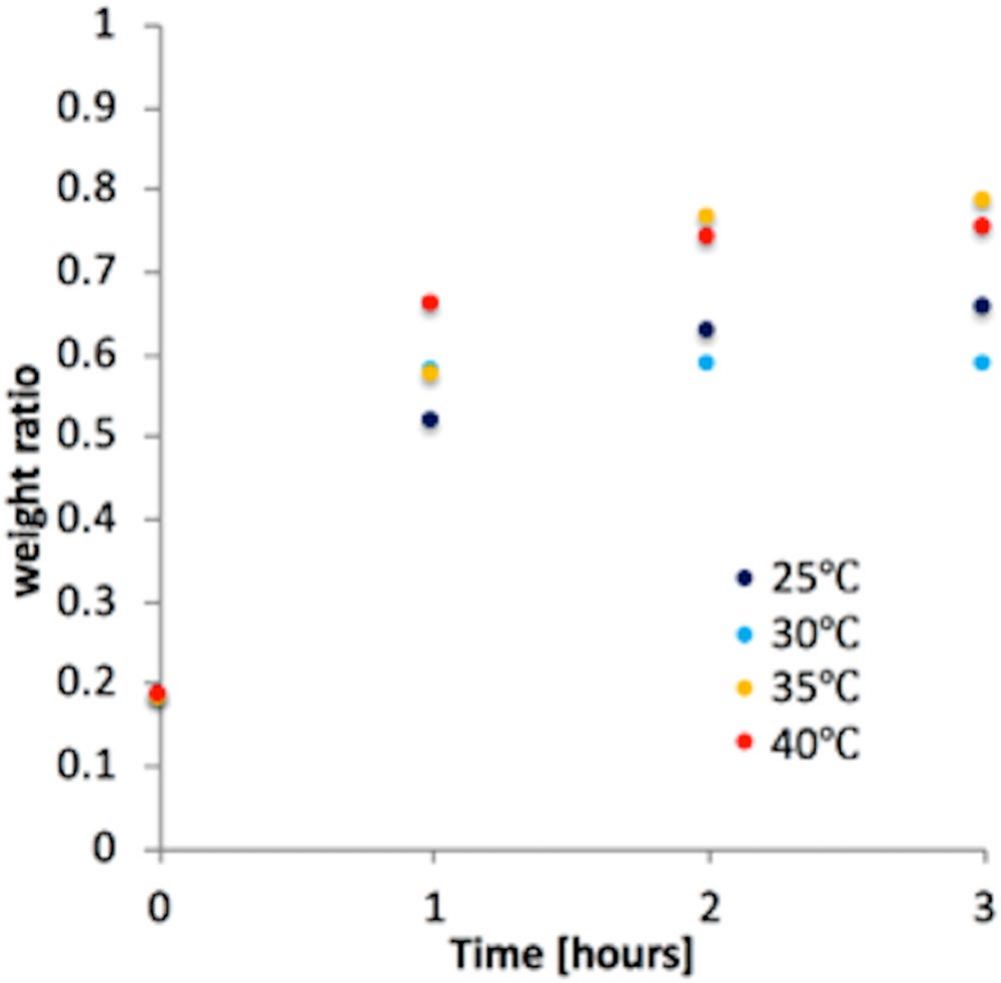
Change in the weight ratio of dried PVA-H specimens during reformation as a function of the time they spent immersed in water; several different temperatures for the water used are shown. A weight ratio of 1 means the weight of the specimen is the same as before drying.

Figure 1 shows the change in the weight ratios of the specimens as a function of the time that the drying process was performed for. The ratios decreased as time increased; the ratios of the specimens with DMSO remained roughly constant at 0.2 after 72 h, while the specimens without DMSO also remained roughly constant at 0.2, but after only 4 h. Figure 2 depicts the change in the weight ratios of the specimens over time after they had reformed following the immersion in water that was maintained at different temperatures. The weight ratios increased over time and remained constant for all of the specimens after 4 h. The weight ratios after 4 h at 25, 30, 35, and 40 °C were 0.825, 0.836, 0.949, and 0.879, respectively.

### Measurements of mechanical properties

**Figure 3:**
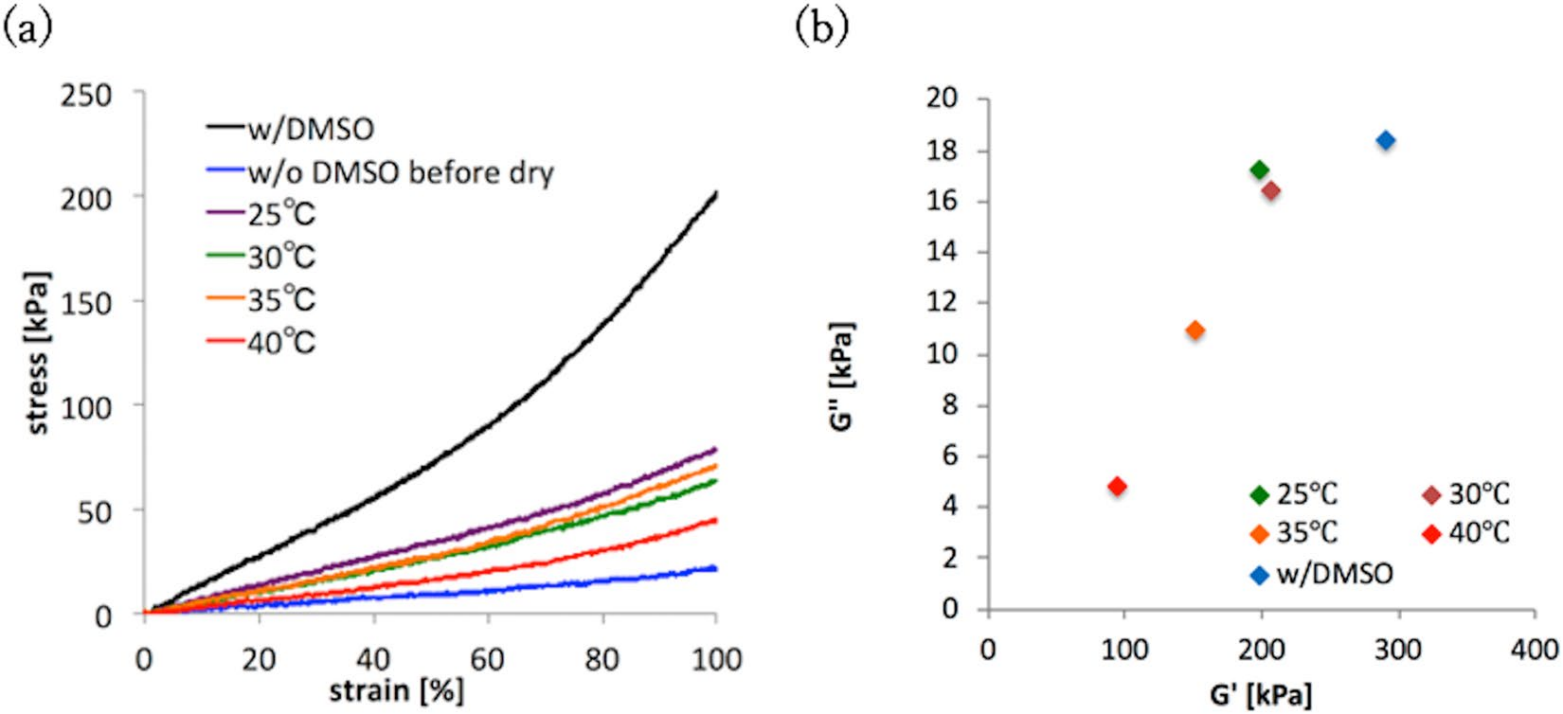
Results of the mechanical property measurements: (**a**) A stress-strain curve obtained from a tensile test conducted for the PVA-H specimens; the figure depicts the results that were obtained before drying and after the reformation of specimens by immersion in water that was maintained at different temperatures. (**b**) Dynamic viscoelasticity of the PVA-H specimens both before and after immersion in water at different temperatures under 1 Hz and a temperature of 23 °C.

Figure 3(a) shows a stress–strain curve of the specimens both before and after they had reformed; results for the PVA-H specimens before both the water immersion and drying processes are also shown in the figure for reference. The Young’s moduli of the reformed PVA-H specimens at 50% (or 0.5) strain were 68.0, 52.6, 55.0, and 32.6 kPa at water immersion temperatures of 25 °C, 30 °C, 35 °C, and 40 °C, respectively. The Young’s modulus of the PVA-H specimen with DMSO before drying at 50% strain was 143.1 MPa, while that of the PVA-H without DMSO before drying was 18.1 kPa.

Figure 3(b) shows the relationship between the dynamic storage and the dynamic loss moduli of every specimen. The dynamic storage moduli of the reformed PVA-H specimens was 198.7, 206.6, 151.4, and 94.59 kPa at water immersion temperatures of 25 °C, 30 °C, 35 °C, and 40 °C, respectively. PVA-H with DMSO before drying was tested as a reference, and it was found to be 291.4 kPa. The dynamic loss moduli of the reformed PVA-H specimens were 17.3, 16.4, 10.9, and 4.85 kPa at water immersion temperatures of 25, 30, 35 and 40 °C, respectively. PVA-H with DMSO before drying was once again used as a reference, and it was found to be 18.4 kPa. Both the storage and loss moduli of the specimens were recovered better by the lower water immersion temperatures in this study.

### XRD measurements

**Figure 4:**
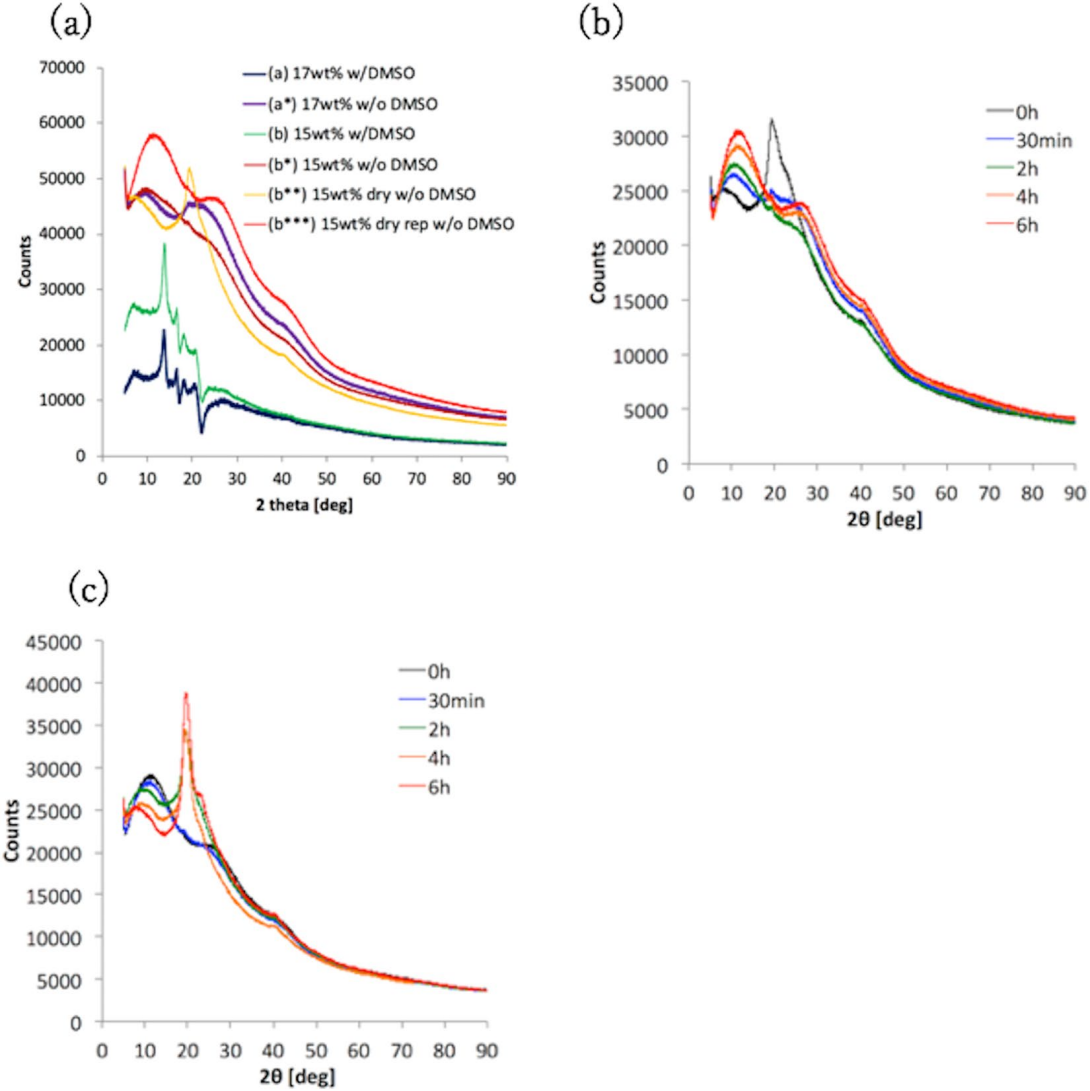
XRD profiles: (**a**) every profile of specimen (**a**) through (b***). (**b**) specimen (b**) in an immersed state. 0 h indicates the time at which the sample was first immersed in 35 °C-water. (**c**) specimen (b*) while it was being dried. 0 h means the time at which drying began at 40 °C.

Figure 4 depicts the XRD profiles of the PVA-H specimens. The profiles of the PP sheet covering the PVA-H specimens were subtracted from the original profiles of the PVA-H specimens with DMSO. The 2θ values at the spectra peak in the specimens were 8.82°, 19.40°, and 11.36° for the PVA-H specimens before they were dried (Fig. 4(a)), for the PVA-H specimens after they had been dried, and for the PVA-H specimens after they had reformed, respectively. The shift in the peak positions of the PVA-H specimens due to these conditions meant that their crystal structures changed both due to the water in them evaporating and due to the water immersion used to reform their original crystal structures. Furthermore, the intensity of the peaks found for the reformed PVA-H specimens was greater than for the PVA-H specimens in the other conditions; this means that the number of crystals in the specimen increased due to the water immersion process. Figure 4(b,c) depict the XRD profiles of the specimens produced using the (b*) and (b**) conditions described in Table 1 while drying and water immersion processes were being performed. These time-lapse measurements indicated that the dried PVA-H specimen (b**) has two spectra peaks, and the 2θ values at the peaks were 8.30° and 19.32° before immersion; the profile around 8.30° was broad, while that around the peak at 19.32° was sharp. After the specimens were immersed in water, the peak at 19.32° completely disappeared, while the peak at 8.30° increased and shifted to 10.92° after being dried for 6 h. Contrastingly, the 2θ value at the peak of the immersed PVA-H specimen was 10.92° before it reformed; this peak decreased and shifted to 7.73° after an immersion of 6 h, and another new peak appeared and increased at approximately 19.40° after the specimen had been immersed for 2 h. These results indicated that the crystals can repeatedly increase and decrease depending on the amount of water in PVA-H.

### Image acquisitions

**Figure 5:**
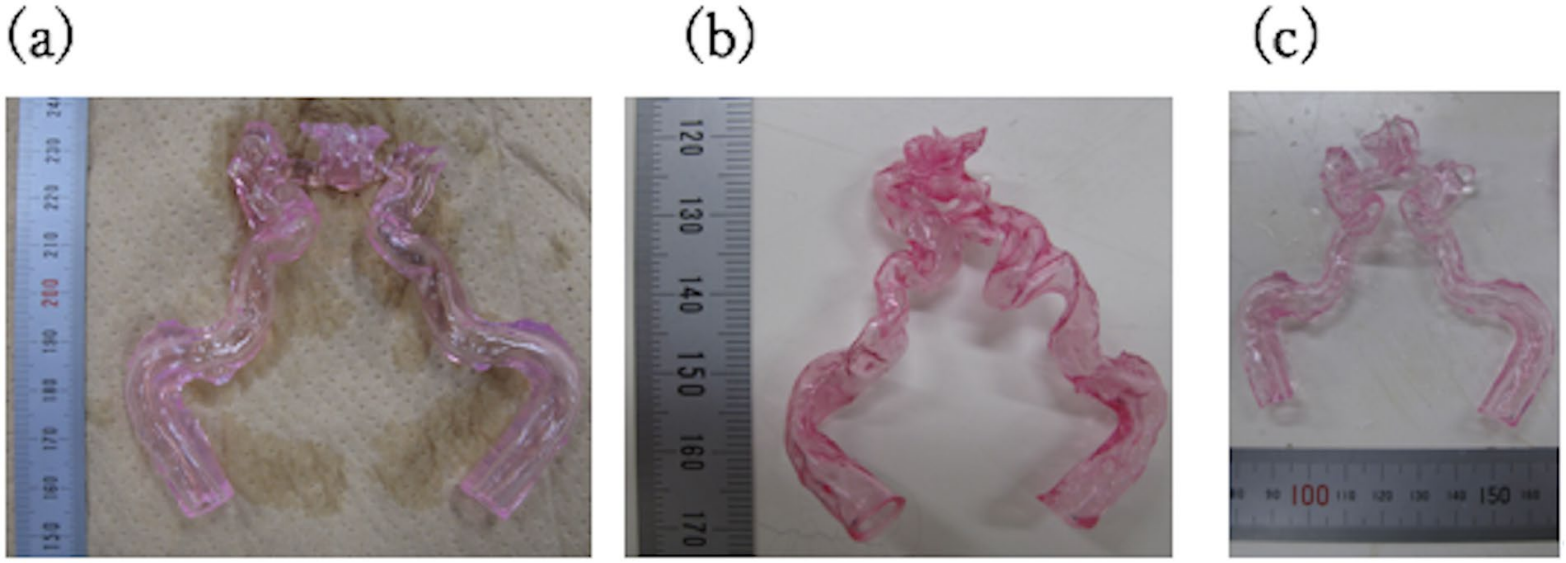
Photographs of the 15 wt% PVA-H blood vessel models: (**a**) before drying, (**b**) after drying, and (**c**) after water immersion.

**Figure 6:**
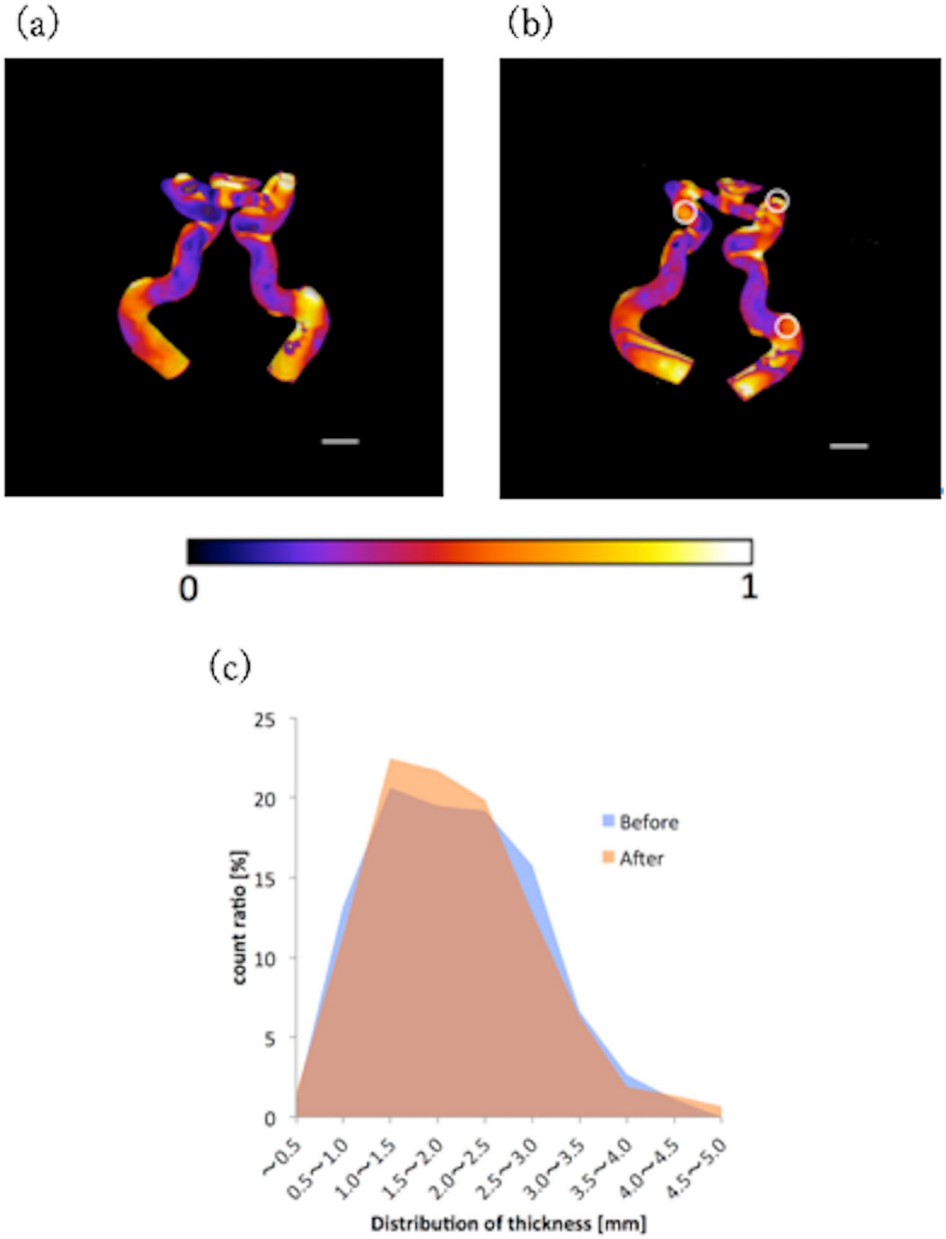
3D reconstruction of the geometry of the blood vessel models with color contours indicating the thicknesses of the models. The thicknesses before and after the reformation processes were analyzed based on the reconstruction data; the data is based on the micro-CT acquired images (**a**) before drying and (**b**) after water immersion. The color contour represents the distribution of the thickness (1 indicates the thickest part (approximately 4.5 mm) in the model), and the white bar in the lower-right-hand-side of the image represents a thickness of 10 mm. (**c**) Histogram of the thicknesses of the blood vessel models both before and after completing the reformation processes.

Figure 5 shows photos of the models. The models completely wrinkled after drying, but after water immersion, the models reformed into their original geometries, although they were now larger in size. Figure 6(a,b) show 3D-reconstructed images developed using the micro-CT acquired images. The color contour represents the distribution of the thickness distribution in every model; the value “1” represents the thickest part in a model. No cracks were found in the models after they had reformed due to water immersion. By comparing the images of the reformed and pre-dried models, we found that the original geometry was preserved after water immersion, though several parts significantly deformed; these are indicated by the white circles in the figure.

Figure 6(c) shows a histogram of the thicknesses of the models; this was used to compare the difference between the thicknesses of the models before and after drying as well as after the model had reformed. From this figure, it can be seen that the thickness of the pre-dried model was preserved even after water immersion. The maximum difference between the thicknesses before and after water immersion was approximately 3.0%, and the thickness of the model in all of the tested conditions remained within 2.5–3.0 mm.

## Discussion

In this study, the influence of drying and reformation processes on the mechanical properties of PVA-H was investigated using the changes in the weight and Young’s modulus of the material. The result of the change in weight during drying indicated that PVA-H without DMSO can be dried quicker and reformed faster than PVA-H with DMSO, even if the time spent being immersed in water is the same. This means that dried PVA-H without DMSO can be reformed sooner than if DMSO is used. It is known that the intermolecular force between DMSO and H_2_O molecules is much greater than that of single molecules such as pure H_2_O or DMSO^17^; the strength of this intermolecular force may prevent water from evaporating quickly, which is why PVA-H that contains DMSO takes a longer time to dry.

The results of the tensile tests and viscoelasticity measurements indicated that the Young’s modulus and dynamic viscoelasticity of reformed PVA-H can be controlled by making sure that the temperature of the water used in the reformation process is within the same range as that found in blood vessels. In addition, the ranges of dynamic viscoelasticity of all of the specimens were also the same as the ranges found by a previous study^4^. These results indicate that the dynamic viscoelasticity properties of PVA-H materials can be retained after drying and water immersion processes have been performed. PVA molecules in crystallites are swollen and dissolve step-by-step based on the increment of the PVA-H temperature^18^. Our results indicate that the swelling of PVA molecules may affect the molecular structure of PVA-H and reproduce its initial Young’s modulus and weight. With regards to the mechanical properties of the tested biomodel, water of 35 °C was found to be effective for the reformation of dried PVA-H, because while water warmer than this can reproduce the weight of the hydrogel, it caused it to lose its Young’s modulus, and the opposite is true when water colder than 35 °C was used. As such, 35 °C water was the best of the temperatures tested for reforming dried PVA-H. However, because the mechanical properties cannot be completely reproduced, as described in the results of the tensile test and the dynamic viscoelasticity measurement, high concentrations of PVA (i.e., more than 15 wt%) should be used in the manufacture of biomodels.

The XRD results for the 2θ value at the peak found by the present study were quite similar to those found by previous studies, regardless of the manufacturing method used^18–20^. Although Otsuka *et al*.^19^ used the scattering vector “*q*” instead of 2θ^19^, this vector can be transformed by Eq. 6, in which λ represents the wavelength:

6 $$q=\frac{4\pi \,\sin \,\theta }{\lambda }$$

This result indicates that the fabrication method used for PVA-H in this study did not affect the crystal structure of PVA-H.

The intensity of the diffraction peak at 19.3° decreases and the peak at 8.3° moves to a higher diffraction angle because of the water immersion (Fig. 4(d)). These results are similar to studies conducted on the influence of temperature on the structure of PVA^18,20^. In these earlier studies, the decrease in intensity and the peak shift caused by the temperature change of samples indicates that the hydrogen bonds and microcrystallites in the PVA-rich phase were gradually being destroyed. This structural deformation occurred due to the variation in the average distance between the microcrystallites, which corresponded to the water content in the specimens. The appearance and disappearance of the peaks at 8.3° and 19.3°, respectively, indicate that the immersed water interposed between the microcrystallites and the intermolecular distance was extended and vice versa. Furthermore, it was discovered that the crystalline fraction decreased when the water content increased^20^. The change in weight of PVA-H during the drying process was due to water evaporation based on an air temperature of 40 °C, and this water evaporation can lead to an increase in the crystalline fraction in PVA-H.

In the results we obtained for the reformation of the dried PVA-H specimens, we found that the reproduction ratios of the Young’s moduli of the specimens between the before and after water immersion conditions were 22.8–47.5%. The XRD measurements indicate that the crystals can reversibly change their structures through the drying and reformation processes. This reversible phenomenon was also observed based on the formation and destruction of hydrogen bonds in a previous study^21^. In addition, Raman spectral analyses from a previous study revealed that the structures of water in PVA-H have an insignificant effect of the fabrication process and depend only on the water content, and that the structure of the polymer network distorts during drying but is not completely reformed by re-swelling^9^. These results support the findings made by both our tensile tests and XRD measurements.

The micro-CT images indicated that the geometry of the models was preserved, in that no cracks formed in them after water immersion. Therefore, the drying process tested is beneficial for model preservation with effectiveness of the model deformation.

The histogram in Fig. 6(c) supports the conclusion that the thickness of the model remains roughly the same after water immersion. This result also indicates that the drying and reformation process used in this study does not change the geometry and mechanical properties of the vessel models tested.

## Conclusions

In this study, a simple reproduction method was developed for the PVA-H blood vessel biomodels. We found that the blood vessel models could be preserved by air-drying, and they could be reformed by immersion in water. The dried model for the human carotid artery can be reproduced, depicting the original geometry and the realistic mechanical properties, three weeks after drying using this method. The XRD measurements indicated that the chemical structure of the PVA-H can change reversibly, depending on the water content. Furthermore, we found that a water temperature of 35 °C was the most advantageous in terms of reproducing both the geometry and the mechanical properties of the dried biomodels; this was determined using micro-CT acquired images, tensile tests, and viscoelastic measurements.

## Electronic supplementary material

Supplemental figure Fig. S1.
