# Supplementary material for: Reproduction method for dried biomodels composed of poly (vinyl alcohol) hydrogels
Source: Sci Rep. 2018 Apr 10;8:5754. doi: 10.1038/s41598-018-24235-z (PMC5893614; doi:10.1038/s41598-018-24235-z)
Supplement: Supplementary file 1 — Supplemental figure Fig. S1. [file 41598_2018_24235_MOESM1_ESM.pdf]

Reproduction method for dried biomodels composed of poly (vinyl alcohol) hydrogels

Yasutomo Shimizu[1*], Narendra Kurnia Putra[2], Makoto Ohta[1]

1. Institute of Fluid Science, Tohoku University
   2-1-1, Katahira, Aoba-ku, Sendai, Miyagi, Japan
2. Graduate School of Engineering, Tohoku University
   6-6, Aramaki-aza-aoba, Aoba-ku, Sendai, Miyagi, Japan

*Corresponding author's email: shimizu@biofluid.ifs.tohoku.ac.jp

(a)

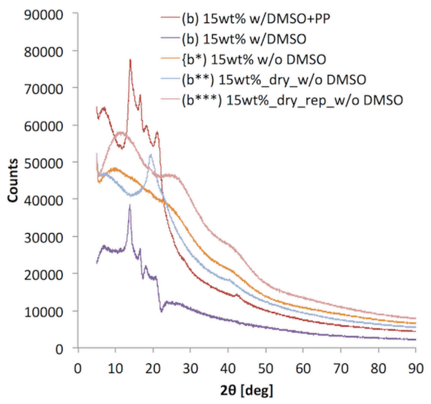

(b)

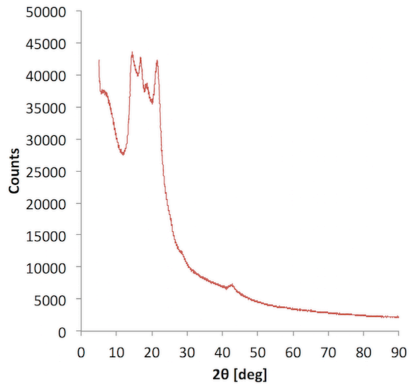

(c)

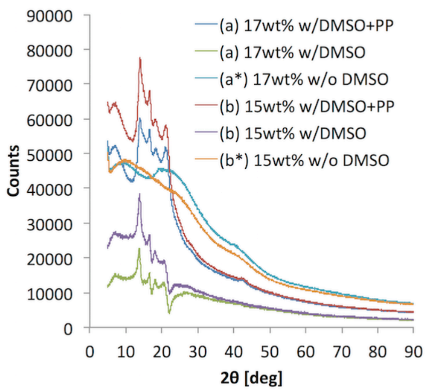

Supplemental figure Fig. S1. XRD profiles: (a) 15 wt% PVA-H specimens during drying. (b) a PP sheet covering PVA-H specimens. (c) comparison of 15 and 17wt% PVA-H specimens (both containing and not containing DMSO).
